# Supplementary material for: Evaluating N95 respirator designs: A mixed-methods pilot and feasibility study
Source: PLoS One. 2025 Dec 3;20(12):e0328746. doi: 10.1371/journal.pone.0328746 (PMC12674537; doi:10.1371/journal.pone.0328746)
Supplement: S1 Table — (DOCX) [file pone.0328746.s001.docx]

**SUPPORTING INFORMATION S1 Table**

**Evaluating N95 Respirator Designs: A Mixed-Methods Pilot and Feasibility Study**

Fatima Sheikh, MS.c^1^, Myrna Dolovich, P.Eng^2,3^, Lisa Schwartz, Ph.D^1^, Sarah Khan, M.D^4,5^, Zeinab Hosseinidoust, Ph.D^6^, and Alison E. Fox-Robichaud, MD.^1,2,5^

1. Department of Health Research Methods, Evidence and Impact, McMaster University, Hamilton, ON, Canada.
2. Department of Medicine, McMaster University, Hamilton, ON, Canada.
3. Department of Pediatrics, McMaster University, Hamilton, ON, Canada.
4. Hamilton Health Sciences, Hamilton, ON, Canada.
5. Department of Chemical Engineering, McMaster University, Hamilton, ON, Canada.

**Corresponding Author:** Dr. Alison-Fox Robichaud

Email: [afoxrob@mcmaster.ca](mailto:afoxrob@mcmaster.ca)

**S1 Table** Objectives, Outcome Measures, and Analysis

| **Objective** | **Outcome** | **Measure of Outcome** |
| --- | --- | --- |
| **Primary Feasibility Objectives** | | |
| 1. Recruitment. | A sample size of 100 HCWs recruited within 4 months and 50% of the participants meet the following criteria:   - Self-identify as non-white - Have at least one of the following characteristics: religious head covering (e.g., Hijab, Turban), glasses and/or facial hair (e.g., beard and/or mustache), and - Identify as female | Count, proportions, and descriptive statistics (where appropriate). |
| 2. Consent | Consent rate of ≥ 80% in approached healthcare workers. | Proportion |
| 3. To perform the PortaCount fit test on all the included participants. | Successful PortaCount Fit Test: Full completion of a fit test or partial completion with a reason why the test was ended early. | Proportion and any changes to the current protocol |
| 4. To collect HCW reported feelings of N95 fit and the impacts of COVID-19 and associated PPE shortages on overall well-being. | Successful HCW-reported data collection: Completion of the survey, defined as at least 80% of the questions have been fully answered. | Proportion |
| **Secondary Objectives** | | |
| 1. To assess outcomes of PortaCount fit test to understand the protection N95s provide in a diverse sample of HCWs | Portacount fit test output (fit factor for all 7 tests and overall fit factor). | Descriptive statistics (mean ± SD) |
| 2. To describe participant-reported measures of N95 fit. | Participant-reported assessment of overall fit, comfort, and breathability. | Description of the results reported, thematic analysis, and. where appropriate, descriptive statistics. |
| 3. To describe any reported negative impacts of the pandemic and the limited availability of N95s on physical and mental well-being of HCWs. | Participant-reported impacts of COVID-19 on their physical and mental wellbeing. |  |

*HCW* Healthcare workers; *COVID-19* Coronavirus Disease 2019; *PPE* Personal Protective Equipment; *SD* Standard Deviation.
