# Supplementary material for: Evaluating N95 respirator designs: A mixed-methods pilot and feasibility study
Source: PLoS One. 2025 Dec 3;20(12):e0328746. doi: 10.1371/journal.pone.0328746 (PMC12674537; doi:10.1371/journal.pone.0328746)
Supplement: S2 Table — (DOCX) [file pone.0328746.s002.docx]

**SUPPORTING INFORMATION S2 Table**

**Evaluating N95 Respirator Designs: A Mixed-Methods Pilot and Feasibility Study**

Fatima Sheikh, MS.c^1^, Myrna Dolovich, P.Eng^2,3^, Lisa Schwartz, Ph.D^1^, Sarah Khan, M.D^4,5^, Zeinab Hosseinidoust, Ph.D^6^, and Alison E. Fox-Robichaud, M.D.^1,2,5^

1. Department of Health Research Methods, Evidence and Impact, McMaster University, Hamilton, ON, Canada.
2. Department of Medicine, McMaster University, Hamilton, ON, Canada.
3. Department of Pediatrics, McMaster University, Hamilton, ON, Canada.
4. Hamilton Health Sciences, Hamilton, ON, Canada.
5. Department of Chemical Engineering, McMaster University, Hamilton, ON, Canada.

**Corresponding Author:** Dr. Alison-Fox Robichaud

Email: [afoxrob@mcmaster.ca](mailto:afoxrob@mcmaster.ca)

| **S2 Table Raw Data** | | | | | | | | | | | | | | | | | | | | | | | | |
| --- | --- | --- | --- | --- | --- | --- | --- | --- | --- | --- | --- | --- | --- | --- | --- | --- | --- | --- | --- | --- | --- | --- | --- | --- |
| **ID** | **Date** | **Sex** | **Gender** | **Ethnicity** | **Conditions of Respirator Use** | **Frequency** | **Duration** | **Previous Fit Test** | **Previous Difficulty Being Fitted** | **Previous Mask Fit** | **Total # of Fit Tests** | **# of Successful Fit Tests** | **# of Unsuccessful Fit Tests** | **Normal Breathing** | **Deep Breathing** | **Head Side to Side** | **Head Up and Down** | **Talking** | **Bending Over** | **Normal Breathing2** | **Final Result** | **Final N95 Fit** | **Menton-Sellion Length** | **Bizygomatic Breadth** |
| 01-001 | 19-Jan | 0 | Male | 6 | 1 |  | 4 | 1 | 0 | 1860s | 1 | 1 | 0 | 200+ | 200+ | 200+ | 200+ | 92 | 168 | 200+ | 168 | 1870+ | 129.12 | 132.84 |
| 01-002 | 28-Jan | 1 | Female | 0 | 1 | 1,2 | 4 | 1 | 0 | 1860S,1870 | 1 | 1 | 0 | 200+ | 200+ | 200+ | 200+ | 200+ | 200+ | 200+ | 200+ | 1870+ | 116.1 | 135.66 |
| 01-003 | 28-Jan | 1 | Female | 2 | 1 | 4 | 4 | 1 | 0 | 1860s | 1 | 1 | 0 | 119 | 200+ | 200+ | 200+ | 200+ | 138 | 200+ | 173 | 1870+ | 105.89 | 128.9 |
| 01-004 | 28-Jan | 1 | Female | 0 | 1 | 1 | 3 | 1 | 0 | 1870 | 1 | 1 | 0 | 200+ | 200+ | 200+ | 200+ | 200+ | 200+ | 200+ | 200+ | 1870+ | 104.94 | 103.87 |
| 01-005 | 28-Jan | 1 | NR | 0 | 1 | NR | 4 | 1 | 0 | 1860 | 1 | 1 | 0 | 200+ | 200+ | 200+ | 200+ | 200+ | 200+ | 200+ | 200+ | 1870+ | 119.04 | 117.33 |
| 01-008 | 15-Feb | 1 | NR | 7 | 1 | 1 | NR | 1 | 1 | NR | 1 | 1 | 0 | 200+ | 200+ | 92 | 200+ | 200+ | 200+ | 200+ | 171 | 1870+ | 132.95 | 120.82 |
| 01-009 | 15-Feb | 1 | Female | 0 | 1 | 1 | 3 | 0 | 0 | 1860s | 2 | 1 | 1 | 200+ | 200+ | 101 | 200+ | 200+ | 200+ | 200+ | 176 | Honeywell DC 365 | 115.11 | 109.39 |
| 01-010 | 15-Feb | 1 | NR | 0 | 1 | 1 | 4 | 1 | 0 | 1860s | 2 | 1 | 1 | 200+ | 200+ | 200+ | 200+ | 200+ | 200+ | 200+ | 200+ | Honeywell DC 365 | 125.16 | 122.61 |
| 01-011 | 15-Feb | 0 | Male | 0 | 1 | 1 | 3 | 1 | 0 | 1860 | 1 | 1 | 0 | 200+ | 200+ | 200+ | 200+ | 200+ | 200+ | 200+ | 200+ | 1870+ | 130.8 | 108.35 |
| 01-012 | 15-Mar | 1 | Female | 0 | 1 | 2 | 2 | 1 | 0 | DC 365 | 1 | 1 | 0 | 200+ | 200+ | 200+ | 53 | 200+ | 200+ | 200+ | 144 | 1870+ | 112.83 | 120.65 |
| 01-013 | 15-Mar | 1 | Female | 0 | 1 | 1 | 3 | 1 | 0 | NR | 1 | 1 | 0 | 200+ | 200+ | 200+ | 195 | 200+ | 200+ | 200+ | 200+ | 1870+ | 116.49 | 104.31 |
| 01-014 | 17-Mar | 1 | Female | 0 | 1 | 1 | 4 | 1 | 0 | DC 365 | 3 | 1 | 2 | 200+ | 200+ | 200+ | 200+ | 200+ | 166 | 132 | 182 | DC 365 | 105.28 | 108.3 |
| 01-015 | 17-Mar | 1 | Female | NR | 1 | 1 | NR | 1 | 0 | NR | 1 | 1 | 0 | 200+ | 200+ | 200+ | 200+ | 200+ | 200+ | 200+ | 200+ | 1870+ | 120.6 | 110.88 |
| 01-016 | 17-Mar | 1 | Female | 0 | 1 | 2 | 2 | 1 | 0 | NR | 1 | 1 | 0 | 200+ | 76 | 85 | 67 | 200+ | 200+ | 156 | 114 | 1870+ | 115.7 | 91.48 |
| 01-017 | 28-Mar | 1 | Female | 0, 1 | 1 | 2 | 3 | 1 | 0 | NR | 3 | 1 | 2 | 200+ | 200+ | 200+ | 200+ | 200+ | 200+ | 162 | 194 | 1860s | 105.36 | 108.1 |
| 01-018 | 28-Mar | 1 | Female | 8 | 1 | 1 | 4 | 1 | 0 | 8210 | 2 | 1 | 1 | 200+ | 200+ | 200+ | 200+ | 200+ | 194 | 68 | 157 | 1870+ | 103.84 | 96.51 |
| 01-019 | 30-Mar | 1 | Female | 11 | 1 | 2 | 4 | 1 | 0 | NR | 3 | 1 | 2 | 200+ | 200+ | 200+ | 92 | 200+ | 200+ | 200+ | 171 | 1804s | 108.33 | 104.62 |
| 01-020 | 30-Mar | 1 | Female | 6 | 1 | 4 | 4 | 1 | 0 | NR | 1 | 1 | 0 | 200+ | 139 | 78 | 96 | 200+ | 200+ | 200+ | 139 | 1870+ | 99.68 | 108.94 |
| 01-021 | 30-Mar | 0 | Male | 8 | 1 | 1 | 3 | 1 | 0 | 1860 | 3 | 1 | 2 | 200+ | 200+ | 200+ | 200+ | 200+ | 200+ | 200+ | 200+ | 1870+ | 131.6 | 121.77 |
| 01-022 | 30-Mar | 1 | Female | 4 | 1 | 4 | 4 | 1 | 0 | 1860s | 3 | 1 | 2 | 137 | 200+ | 92 | 200+ | 200+ | 70 | 200+ | 134 | 1804s | 88.48 | 103.31 |
| 01-023 | 30-Mar | 1 | NR | 0 | 1 | 3 | 2 | 1 | 0 | 1860 | 1 | 1 | 0 | 200+ | 200+ | 200+ | 200+ | 200+ | 200+ | 200+ | 200+ | 1870+ | 119.93 | 108.26 |
| 01-024 | 31-Mar | 0 | Male | 2 | 1 | 4, As needed | 4 | 1 | 0 | 8210 | 1 | 1 | 0 | 200+ | 200+ | 200+ | 197 | 181 | 61 | 94 | 133 | 1870+ | 113.62 | 124.77 |
| 01-025 | 31-Mar | 1 | Female | 0, 7 | 1 | 4, As needed | 4 | 1 | 0 | NR | 2 | 1 | 1 | 200+ | 123 | 200+ | 168 | 200+ | 200+ | 200+ | 179 | 1870+ | 104.34 | 105.54 |
| 01-026 | 31-Mar | 1 | Female | 0 | 1 | 2 | 3 | 1 | 0 | 1860s | 1 | 1 | 0 | 200+ | 200+ | 200+ | 65 | 150 | 79 | 200+ | 128 | 1870+ | 103.5 | 98.11 |
| 01-027 | 31-Mar | 1 | Female | 2 | 1 | 2 | 4 | 1 | 0 | 1860s | 1 | 1 | 0 | 200+ | 200+ | 200+ | 37 | 147 | 200+ | 125 | 114 | 1870+ | 103.11 | 95.83 |
| 01-028 | 06-Apr | 1 | Female | 0 | 1 | 2 | 2 | 0 | 0 | NR | 3 | 1 | 2 | 200+ | 200+ | 200+ | 200+ | 200+ | 200+ | 200+ | 200+ | 1804s | 109.39 | 109.87 |
| 01-029 | 08-Apr | 1 | Female | 11 | 1 | 1 | 4 | 1 | 0 | NR | 1 | 1 | 0 | 200+ | 200+ | 200+ | 175 | 200+ | 200+ | 172 | 192 | 1870+ | 123.59 | 99.88 |
| 01-030 | 11-Apr | 1 | Female | 0 | 1 | 4 | 4 | 1 | 0 | NR | 1 | 1 | 0 | 200+ | 200+ | 200+ | 200+ | 200+ | 200+ | 200+ | 200+ | 1870+ | 124 | 108.05 |
| 01-031 | 14-Apr | 1 | Female | 6 | 1 | 1 | 4 | 1 | 0 | NR | 1 | 1 | 0 | 200+ | 200+ | 200+ | 200+ | 200+ | 200+ | 200+ | 200+ | DC 365 | 109.39 | 109.01 |
| 01-032 | 20-Apr | 1 | NR | 0 | 1 | 2 | 3 | 1 | 0 | 8210 | 1 | 1 | 0 | 181 | 146 | 100 | 111 | 155 | 126 | 144 | 133 | 1870+ | 110.85 | 93.34 |
| 01-033 | 20-Apr | 1 | Female | 0 | 1 | 4 | 4 | 1 | 0 | 1860 | 1 | 1 | 0 | 200+ | 200+ | 188 | 200+ | 90 | 144 | 176 | 159 | 1870+ | 120.19 | 117.82 |
| 01-034 | 27-Apr | 0 | Male | 0 | 1 | 1 | 2 | 1 | 0 | 8210 | 1 | 1 | 0 | 200+ | 200+ | 200+ | 200+ | 200+ | 200+ | 200+ | 200+ | 1870+ | 118.12 | 117.39 |
| 01-035 | 27-Apr | 1 | NR | 6 | 1 | 1 | 3 | 1 | 0 | 1860s | 1 | 1 | 0 | 150 | 200+ | 200+ | 200+ | 200+ | 57 | 62 | 117 | 1870+ | 109.95 | 109.33 |
| 01-036 | 28-Apr | 1 | Female | 0 | 1 | 4, As needed | 2 | 1 | 1 | DC 365 | 5 | 1 | 4 | 17918 | 14561 | 501 | 6838 | 8680 | 20998 | 45591 | 2856 | P100 (medium), 6200 series | 100.03 | 109.05 |
| 01-037 | 05-May | 1 | Female | 0 | 1 | 1 | 4 | 1 | 0 | 1804S | 2 | 1 | 1 | 200+ | 200+ | 200+ | 86 | 200+ | 173 | 200+ | 165 | 1870+ | 107.94 | 118.11 |
| 01-038 | 05-May | 0 | Male | 10 | 1 | 1 | 4 | 1 | 0 | NR | 4 | 1 | 3 | 200+ | 200+ | 200+ | 200+ | 200+ | 200+ | 200+ | 200+ | 1870+ | 113.93 | 126.48 |

**Sex:** 0 = male, 1 = female, 111 = no answer

**Ethnicity:** 0 = White (Caucasian), 1 = Indigenous, 2 = Chinese, 3 = Korean, 4 = South East Asian, 5 = Arab/West Asian, 6 = Filipino, 7 = Latin American, 8 = Black, 9 = Japanese, 10 = South Asian, 11 = Self-identify, 111 = No answer.

**Conditions of respirator use**: 1 = Airborne Isolation, 2 = Dust, 3 = Other.

**Frequency of use:** 1 = Daily, 2 = Weekly, 3 = Monthly, 4 = Other.

**Duration of use:** 1 = < 15min, 2 = > 15min, 3 = > 2 hours, 4 = Variable, 5 = Other

**Previous fit test:** 0 = No, 1 = Yes

**Previous difficulty being fit tested:** 0 = No, 1 = Yes
