# Supplementary material for: Evaluating N95 respirator designs: A mixed-methods pilot and feasibility study
Source: PLoS One. 2025 Dec 3;20(12):e0328746. doi: 10.1371/journal.pone.0328746 (PMC12674537; doi:10.1371/journal.pone.0328746)
Supplement: S1 File — (DOCX) [file pone.0328746.s003.docx]

**SUPPORTING INFORMATION S1 File**

**Evaluating N95 Respirator Designs: A Mixed-Methods Pilot and Feasibility Study**

Fatima Sheikh, MS.c^1^, Myrna Dolovich, P.Eng^2,3^, Lisa Schwartz, Ph.D^1^, Sarah Khan, M.D^4,5^, Zeinab Hosseinidoust, Ph.D^6^, and Alison E. Fox-Robichaud, M.D^1,2,5^

1. Department of Health Research Methods, Evidence and Impact, McMaster University, Hamilton, ON, Canada.
2. Department of Medicine, McMaster University, Hamilton, ON, Canada.
3. Department of Pediatrics, McMaster University, Hamilton, ON, Canada.
4. Hamilton Health Sciences, Hamilton, ON, Canada.
5. Department of Chemical Engineering, McMaster University, Hamilton, ON, Canada.

**Corresponding Author:** Dr. Alison-Fox Robichaud

Email: [afoxrob@mcmaster.ca](mailto:afoxrob@mcmaster.ca)

**S1 File** N95 Fit Survey Design

The survey was designed to gauge the HCW's assessment of (1) fit, comfort, and breathability of N95s and (2) the impact of PPE-related challenges on their physical and mental health, using open and closed-ended questions. The survey was divided into three parts that included participant demographics, fit and breathability of N95s and surgical masks, and physical and mental health. The demographic section included sex, gender, ethnicity, presence of headwear (e.g., hijab or turban), facial hair and glasses. The definitions of sex, gender, and ethnicity were included in the survey to facilitate accurate responses. The HCWs were able to select more than one ethnicity or self-identify if the included categories did not reflect how they would identify. For the remaining 2 sections of the survey, closed-ended questions on N95 and surgical mask fit, comfort, and breathability, as well as PPE-related impacts on the physical and mental health of HCWs, were evaluated using 5-point Likert scales. Each question or series of related questions was followed by open-ended questions to collect qualitative, HCW-reported experiences of N95 fit to supplement the results of the closed-ended questions, identify additional information not captured in the closed-ended questions, and to assess the feasibility of collecting in-depth qualitative data.

Domains, including gender, ethnicity, physical and mental well-being, were included based on frequently reported challenges in the literature^1,2^, and the ongoing impacts of the COVID-19 pandemic on the overall well-being of front-line HCWs. However, to assess the suitability of the questions, readability, and overall clarity, the survey was pilot tested in a sample of critical care healthcare workers (n = 3). Minor changes were made to improve the clarity of the questions. No questions were added or removed, and no other significant changes were made.

The results of surgical mask fit, comfort, and breathability are not reported in this manuscript.

**Part I. Participant Demographics**

| **Please select the appropriate response for each question.** | |
| --- | --- |
| **1. Occupation**  (e.g., staff physician, nurse, resident etc.) | ______________*_______________*   \| ☐ \| Prefer not to answer \| \| --- \| --- \| |
| **2. Sex** Sex (biological) refers to the sex assigned at birth. Sex is typically assigned based on a person's reproductive system. ^3^ | \| ☐ \| Male \| \| --- \| --- \| \| ☐ \| Female \| \| ☐ \| Prefer not to answer \| |
| **3. Gender** Gender refers to the gender that a person internally feels ('gender identity' along the gender spectrum) and/or the gender a person publicly expresses ('gender expression') in their daily life.^4^ | \| ______________*_______________* \| \| \| --- \| --- \| \| ☐ \| Prefer not to answer \| |
| **4. Ethnicity.** Please select **all** that apply.  Ethnic origin refers to the ethnic or cultural origins of a person’s ancestors.^5,6^ Ethnicity consists of the cultural characteristics that identify a person as belonging to a particular group.^7^ | |
| \| ☐ \| Indigenous (e.g., First Nations, Inuit and Métis) \| ☐ \| Arab/West Asian (e.g., Armenian, Egyptian, Iranian, Lebanese, Moroccan) \| ☐ \| Black (e.g., African, Haitian, Jamaican, Somali) \| \| --- \| --- \| --- \| --- \| --- \| --- \| \| ☐ \| Chinese \| ☐ \| Filipino \| ☐ \| Japanese \| \| ☐ \| Korean \| ☐ \| Latin American \| ☐ \| South Asian \| \| ☐ \| South East Asian \| ☐ \| White (Caucasian) \| ☐ \| Prefer to self-identify:  ___________________ \| \| ☐ \| Prefer not to answer \| \| \| \| \| | |

**5. Date of Birth** (YYYY-MM-DD): __ __ __ __- __ __ - __ __

The purpose of the following question is to determine which, if any, of the following characteristics may/have had an impact on the fit or comfort of **N95 respirators** and/or **surgical masks.**

**6. Please select all the characteristics that apply to you.**

|  | **Yes** | **No** | |
| --- | --- | --- | --- |
| A. Religious head covering (e.g., Hijab, Turban) | ☐ | ☐ | |
| B. Glasses | ☐ | ☐ | |
| C. Facial Hair | ☐ | ☐ | |
| D. Other characteristics (that you feel have impacted the fit) | ☐ | ☐ | |
| Please specify any additional characteristics here: | _______________________________________ | | |
| E. Prefer not to answer | ☐ | |  |

**Part II. N95 Respirator & Surgical Mask Fit and Breathability**

**N95 Respirators**

| **Please select the appropriate response for each question.** | |
| --- | --- |
| 7. Have you previously been fit tested for an N95 respirator? | \| ☐ \| Yes \| \| --- \| --- \| \| ☐ \| No \| |

|  | **Strongly Disagree** | **Disagree** | **Neutral** | | **Agree** | **Strongly Agree** | | **Not applicable** | |
| --- | --- | --- | --- | --- | --- | --- | --- | --- | --- |
| 8. **N95 respirators** fit me well. | ☐ | ☐ | | ☐ | ☐ | | ☐ | | ☐ |
| 9. **N95 respirators** are comfortable. | ☐ | ☐ | | ☐ | ☐ | | ☐ | | ☐ |
| 10**. N95 respirators** are breathable. | ☐ | ☐ | | ☐ | ☐ | | ☐ | | ☐ |

11. Based on your experiences of wearing an **N95 respirator**, please describe any challenges you have encountered regarding the fit, comfort, and breathability.

|  |
| --- |

**Surgical Masks**

|  | **Strongly Disagree** | **Disagree** | **Neutral** | **Agree** | **Strongly Agree** | **Not Applicable** |
| --- | --- | --- | --- | --- | --- | --- |
| 12. **Surgical masks** fit me well | ☐ | ☐ | ☐ | ☐ | ☐ | ☐ |
| 13. **Surgical masks** are comfortable | ☐ | ☐ | ☐ | ☐ | ☐ | ☐ |
| 14**. Surgical masks** are breathable | ☐ | ☐ | ☐ | ☐ | ☐ | ☐ |

15. Based on your experiences of wearing a **surgical mask**, please describe any challenges you have encountered regarding the fit, comfort, and breathability.

|  |
| --- |

**Part III. Physical and Mental Health**

The following section is designed to better understand your experiences with wearing masks and N95 respirators for extended periods of time, and how this has impacted your physical and/or mental health, if at all. After each set of questions (one each for physical and mental health), there are sections for you to share, in your own words, the impacts and any experiences that may not have captured in the questions.

| 16. On a scale of 1 to 10, with 1 being ‘poor’ and 10 being ‘excellent’, please rate your **overall** experience wearing an N95 respirator. | | | | | | | | | |
| --- | --- | --- | --- | --- | --- | --- | --- | --- | --- |
| 1 | 2 | 3 | 4 | 5 | 6 | 7 | 8 | 9 | 10 |
| **Poor** |  |  |  |  |  |  |  |  | **Excellent** |

**Physical Health**

| 17. When wearing an N95, have you experienced any **physical** discomfort? | \| ☐ \| Yes \| \| --- \| --- \| \| ☐ \| No \| \| ☐ \| Prefer not to answer \| |
| --- | --- | --- | --- | --- | --- | --- | --- |

18. If so, please specify which of the following **physical** discomforts you have experienced. Select all that apply.

|  | **Yes** | **No** |
| --- | --- | --- |
| A. Pressure/Pain | ☐ | ☐ |
| B. Itching | ☐ | ☐ |
| C. Nausea | ☐ | ☐ |
| D. Dizziness | ☐ | ☐ |
| E. Headaches | ☐ | ☐ |
| F. Other | ☐ | ☐ |
| Please specify any additional discomforts here: | __________________________________ | |
| G. Prefer not to answer | ☐ |  |

19. Are there any other **physical discomforts/symptoms** you have experienced either more frequently or directly as a result of wearing an N95 respirator for extended periods of time?

|  |
| --- |

**For each of the following statements, please select the most appropriate response.**

|  | **Strongly Disagree** | | **Disagree** | | **Neutral** | | | **Agree** | **Strongly Agree** | **Prefer not to answer** |
| --- | --- | --- | --- | --- | --- | --- | --- | --- | --- | --- |
| 20. The use of PPE for prolonged periods of time at has negatively impacted my **mental health**. | | ☐ | | ☐ | | | ☐ | ☐ | ☐ | ☐ |
| 21. Limited access to appropriate PPE/PPE shortages have negatively affected my **mental health**. | | ☐ | | ☐ | | ☐ | | ☐ | ☐ | ☐ |
| 22. The fit of currently available N95s has negatively affected my **mental health**. | | ☐ | | ☐ | | ☐ | | ☐ | ☐ | ☐ |

| 23. Are there any other factors or experiences, related to the use of an N95 respirator, that you feel have impacted your **mental health**? | \| ☐ \| Yes \| \| --- \| --- \| \| ☐ \| No \| \| ☐ \| Prefer not to answer \| |
| --- | --- | --- | --- | --- | --- | --- | --- |

24. If you selected “Yes” to the above question, please describe the factors and/or experiences that have impacted your mental well-being in the box below.

|  |
| --- |

| 25. Are there other factors related to the use of N95 respirators and/or experiences related to the use of N95s, during the COVID-19 pandemic, that have negatively impacted your physical and/or mental well-being? | \| ☐ \| Yes \| \| --- \| --- \| \| ☐ \| No \| \| ☐ \| Prefer not to answer \| |
| --- | --- | --- | --- | --- | --- | --- | --- |

If so, please specify in the space provided below:

|  |
| --- |

26. Today’s Date (YYYY/MM/DD): __ __ __ __- __ __ - __ __

Thank you for taking the time to fill out this survey! If you have any additional questions or concerns regarding the study, please contact the Principal Investigator, Dr. Fox-Robichaud at [afoxrob@mcmaster.ca](mailto:afoxrob@mcmaster.ca), or Fatima Sheikh, at [sheikf9@mcmaster.ca](mailto:sheikf9@mcmaster.ca).

**References**

1. Wardhan R, Brennan MM, Brown HL, Creech TB. Does a Modified Adhesive Respirator Improve the Face Seal for Health Care Workers Who Previously Failed a Fit Test?: A Pilot Study During the Coronavirus Disease 2019 Pandemic. *Aa Pract*. 2020;14(8):e01264. doi:10.1213/XAA.0000000000001264

2. Green S, Gani A, Bailey M, Brown O, Hing CB. Fit-testing of respiratory protective equipment in the UK during the initial response to the COVID-19 pandemic. *J Hosp Infect*. 2021;113:180-186. doi:10.1016/j.jhin.2021.04.024

3. Government of Canada SC. Sex of person. Published February 1, 2018. Accessed March 16, 2021. https://www23.statcan.gc.ca/imdb/p3Var.pl?Function=DEC&Id=24101

4. Government of Canada SC. Classification of gender - M - Male gender. Published February 1, 2018. Accessed October 16, 2020. https://www23.statcan.gc.ca/imdb/p3VD.pl?Function=getVD&TVD=467245&CVD=467245&CLV=0&MLV=1&D=1

5. Government of Canada SC. Ethnic origin of person. Published December 2, 2015. Accessed March 18, 2021. https://www23.statcan.gc.ca/imdb/p3Var.pl?Function=DEC&Id=103475

6. Government of Canada SC. Previous standard - Race (ethnicity). Published July 15, 1998. Accessed March 16, 2021. https://www.statcan.gc.ca/eng/concepts/definitions/previous/preethnicity

7. Robinson A. Race vs. Ethnicity vs. Nationality: All You Need to Know. Accessed March 18, 2021. https://blog.prepscholar.com/race-vs-ethnicity-vs-nationality
