# Supplementary material for: Evaluating N95 respirator designs: A mixed-methods pilot and feasibility study
Source: PLoS One. 2025 Dec 3;20(12):e0328746. doi: 10.1371/journal.pone.0328746 (PMC12674537; doi:10.1371/journal.pone.0328746)
Supplement: S2 File — (DOCX) [file pone.0328746.s004.docx]

**SUPPORTING INFORMATION S2 File**

**Evaluating N95 Respirator Designs: A Mixed-Methods Pilot and Feasibility Study**

Fatima Sheikh, MS.c^1^, Myrna Dolovich, P.Eng^2,3^, Lisa Schwartz, Ph.D^1^, Sarah Khan, M.D^4,5^, Zeinab Hosseinidoust, Ph.D^6^, and Alison E. Fox-Robichaud, M.D^1,2,5^

1. Department of Health Research Methods, Evidence and Impact, McMaster University, Hamilton, ON, Canada.
2. Department of Medicine, McMaster University, Hamilton, ON, Canada.
3. Department of Pediatrics, McMaster University, Hamilton, ON, Canada.
4. Hamilton Health Sciences, Hamilton, ON, Canada.
5. Department of Chemical Engineering, McMaster University, Hamilton, ON, Canada.

**Corresponding Author:** Dr. Alison-Fox Robichaud

Email: [afoxrob@mcmaster.ca](mailto:afoxrob@mcmaster.ca)

**S2 File** Study Amendment

Study recruitment began on January 4^th^ at Hamilton General Hospital (HGH). However, due to the significant strain on the fit test clinic (i.e., wait times **≥** an hour), no participants were recruited until January 14^th^. Following two months of very minimal recruitment, a study amendment was submitted to the HiREB on February 27^th^, to expand the eligible sites. This amendment was designed to increase study recruitment by addressing (1) the strain on the fit test clinic, (2) the number of days the fit test clinic was run (on average only 1 day/week at HGH), and (3) the limited staff qualified to perform a PortaCount Fit test. Ultimately, the amendment was necessary in large part due to the challenges associated with the COVID-19 pandemic in hospital settings, and by extension the challenges of conducting research in this context. The amendment was approved on March 6^th^, and the last participant was recruited on May 6^th^.
