# Supplementary material for: Evaluating N95 respirator designs: A mixed-methods pilot and feasibility study
Source: PLoS One. 2025 Dec 3;20(12):e0328746. doi: 10.1371/journal.pone.0328746 (PMC12674537; doi:10.1371/journal.pone.0328746)
Supplement: S3 File — (DOCX) [file pone.0328746.s005.docx]

**SUPPORTING INFORMATION S3 File**

**Evaluating N95 Respirator Designs: A Mixed-Methods Pilot and Feasibility Study**

Fatima Sheikh, MS.c^1^, Myrna Dolovich, P.Eng^2,3^, Lisa Schwartz, Ph.D^1^, Sarah Khan, M.D^4,5^, Zeinab Hosseinidoust, Ph.D^6^, and Alison E. Fox-Robichaud, M.D^1,2,5^

1. Department of Health Research Methods, Evidence and Impact, McMaster University, Hamilton, ON, Canada.
2. Department of Medicine, McMaster University, Hamilton, ON, Canada.
3. Department of Pediatrics, McMaster University, Hamilton, ON, Canada.
4. Hamilton Health Sciences, Hamilton, ON, Canada.
5. Department of Chemical Engineering, McMaster University, Hamilton, ON, Canada.

**Corresponding Author:** Dr. Alison-Fox Robichaud

Email: [afoxrob@mcmaster.ca](mailto:afoxrob@mcmaster.ca)

**S3 File** Additional Results of the Qualitative Survey

In addition to the experiences of wearing N95s, and the perceived fit, comfort, and breathability of respirators, the impact of these experiences on HCWs mental health was also assessed. 8 (22.2%) of the 36 HCWs reported negative impacts due to prolonged use of N95s, 13 (36.1%) reported negative impacts due to limited access during the early stages of the pandemic, and 4 (11.1%) reported negative impacts on their mental health due to the fit of N95s. Although the number of responses to the open-ended questions related to mental health were limited, HCWs reported the following experiences as having negative impacts to their mental health and well-being:

“Ability to communicate with patients – unable to fully understand some patients while wearing a mask – I listen with my eyes.”

“Very uncomfortable. Left to debate whether to wear mask (N95 – 1860s) + be in pain or to protect mental health at work. I feel like we shouldn’t have to choose.”

“Worrying that the N95 not working or leaking and potentially getting COVID-19,”
