# Supplementary material for: Evaluating N95 respirator designs: A mixed-methods pilot and feasibility study
Source: PLoS One. 2025 Dec 3;20(12):e0328746. doi: 10.1371/journal.pone.0328746 (PMC12674537; doi:10.1371/journal.pone.0328746)
Supplement: Attachment — Submitted filename: Reviewers comments-PONE-D-25-34031.docx [file pone.0328746.s006.docx]

**Letter to the Editor (Academic Peer Review)**

**To:**
The Editor, *PLOS ONE*
**Re:** Manuscript ID: PONE-D-25-34031
**Title:** *Evaluating N95 Respirator Designs: A Mixed-Methods Pilot and Feasibility Study*

Dear Editor,

I would like to sincerely thank you for inviting me to review the manuscript titled Manuscript ID: PONE-D-25-34031, *Evaluating N95 Respirator Designs: A Mixed-Methods Pilot and Feasibility Study*". I appreciate the opportunity to contribute to the peer-review process and support the high standards of PLOS ONE.

I have reviewed the above manuscript, which addresses an important and timely question about the fit, comfort, and breathability of N95 respirators among healthcare workers of diverse genders and ethnicities. The work is relevant to occupational health, equity in PPE design, and pandemic preparedness. The mixed-methods approach is appropriate and offers valuable context to the quantitative fit test results.

However, several areas would benefit from clarification and additional detail before the manuscript can be considered for publication. My major concerns relate to:

**Comment 1:** The integration of qualitative and quantitative results, which is mentioned but not described in enough methodological detail.

**Comment 2:** The aggregation of all non-White participants into one group, which may obscure important differences.

**Comment 3:** Limited discussion of the implications of the feasibility shortfall, both methodologically and in terms of generalizability.

**Comment 4:** Greater contextualization of the “out-of-range” anthropometric finding from Figure 2.

Below, I provide detailed comments, embedded within the manuscript text for ease of reference.

Sincerely,
Anahita Fakherpour

**Embedded Reviewer Comments in Manuscript**

**Abstract (lines 23–48)**

**Comment 1:** Clarify why the feasibility target was set at 100 participants, and what the reduced sample size means for interpreting both quantitative and qualitative results. Consider adding a sentence on whether this impacts the power to detect differences.

**Background (lines 62–96)**

**Comment 2:** The background section is strong, but the reliance on pre-pandemic anthropometric data references (e.g., 2005) should be supplemented with more recent post-COVID PPE design literature.
**Comment 3:** If any Canadian-specific anthropometric studies exist, briefly mention them to strengthen the rationale for conducting this work in Canada.

**Methods (lines 106–198)**

**Comment 4:** More detail is needed on how the “statistics-by-theme joint display” was created—did integration involve iterative coding, and how were disagreements resolved?
**Comment 5:** Justify the requirement that 50% of participants be non-White or have other specific characteristics. Was this based on effect size estimation, diversity representation targets, or prior literature?
**Comment 6:** Explain why no exploratory non-parametric statistics (e.g., Mann–Whitney U tests) were used, as these can be informative even in small samples.
**Comment 7:** Indicate whether fit testers were blinded to participant ethnicity and gender to reduce potential observer bias.

**Comment 8:** The exclusion criteria should be reported precisely.

**Results – Feasibility (lines 200–231)**

**Comment 9:** The feasibility shortfall is described, but more interpretation is needed. Were these challenges purely pandemic-related, or do they indicate systemic issues in recruiting HCWs for PPE research?
**Comment 10:** Consider quantifying the recruitment rate per week before and after the study amendment to better illustrate the impact of additional sites.

**Results – Quantitative Fit Test (lines 233–259)**

**Comment 11:** For anthropometric results, consider providing percentage differences to make the magnitude of differences more interpretable.
**Comment 12:** The finding that 75% of participants fall outside the NIOSH bivariate panel range is important—state briefly in Results what this implies for respirator design testing standards.

**Results – Qualitative (lines 261–297)**

**Comment 13:** Tables 4a and 4b should include scale anchors (e.g., 0 = poor, 4 = excellent) directly in the table for clarity.
**Comment 14:** In reporting comfort and breathability scores, you may wish to highlight how these subjective measures aligned—or conflicted—with quantitative fit factors.

**Discussion (lines 299–360)**

**Comment 15:** The comparison to prior literature is solid, but the discussion should explicitly address whether the trends observed (e.g., gender/ethnic disparities in fit) are likely to persist outside pandemic surge conditions.
**Comment 16:** Acknowledge the absence of participants with religious head coverings or facial hair, as this limits the generalizability to all HCW subgroups.

**Conclusion (lines 362–368)**

**Comment 17:** Strengthen the policy recommendations by explicitly calling for updates to the NIOSH bivariate panels and Canadian standards to incorporate gender and ethnic diversity in respirator design, along with specific recommendations for manufacturers.

**Figures and Tables**

**Comment 18:** Include sample sizes (n) in figure legends for transparency.
**Comment 19:** In Figure 2, annotate the proportion of participants outside the panel range for emphasis.
