# Supplementary material for: Evaluating N95 respirator designs: A mixed-methods pilot and feasibility study
Source: PLoS One. 2025 Dec 3;20(12):e0328746. doi: 10.1371/journal.pone.0328746 (PMC12674537; doi:10.1371/journal.pone.0328746)
Supplement: Attachment — Submitted filename: Response to Reviewers.docx [file pone.0328746.s008.docx]

**JOURNAL REQUIREMENTS**

Please ensure that your manuscript meets PLOS ONE's style requirements, including those for file naming. The PLOS ONE style templates can be found at

<https://journals.plos.org/plosone/s/file?id=wjVg/PLOSOne_formatting_sample_main_body.pdf>

[https://journals.plos.org/plosone/s/file?id=ba62/PLOSOne_formatting_sample_title_authors_affiliations.pdf](https://track.editorialmanager.com/CL0/https:%2F%2Fjournals.plos.org%2Fplosone%2Fs%2Ffile%3Fid=ba62%2FPLOSOne_formatting_sample_title_authors_affiliations.pdf/1/010f0198c1ba1e1d-095d31c6-14c1-4a8e-b578-bfc0588f2398-000000/Yfi130OGbKpSF9bE9KgTEfBJ-M8AQT124l4OOMuATWY=224)

**Response:** Thank you. We have re-formatted our manuscript to align with PLOS One’s style requirements.

Please note that funding information should not appear in any section or other areas of your manuscript. We will only publish funding information present in the Funding Statement section of the online submission form. Please remove any funding-related text from the manuscript.

**Response:** We have removed all funding information from the manuscript.

Thank you for stating the following financial disclosure:

“This study was funded by the Canadian Institute for Health Research (CIHR) (202005VR4-447804-CCF) for one year beginning June 1st, 2020. FS was also supported by an Ontario Graduate Fellowship Award during the conduct of this study.”

Please state what role the funders took in the study.  If the funders had no role, please state: "The funders had no role in study design, data collection and analysis, decision to publish, or preparation of the manuscript."

If this statement is not correct you must amend it as needed.

Please include this amended Role of Funder statement in your cover letter; we will change the online submission form on your behalf.

**Response:** We have added a statement indicating the funder had no role in any of the study proceedings and included a revised statement in our cover letter.

4. We note that your Data Availability Statement is currently as follows: All relevant data are within the manuscript and in Supporting Information files.

Please confirm at this time whether or not your submission contains all raw data required to replicate the results of your study. Authors must share the “minimal data set” for their submission. PLOS defines the minimal data set to consist of the data required to replicate all study findings reported in the article, as well as related metadata and methods (<https://journals.plos.org/plosone/s/data-availability#loc-minimal-data-set-definition>).

For example, authors should submit the following data:

- The values behind the means, standard deviations and other measures reported;

- The values used to build graphs;

- The points extracted from images for analysis.

Authors do not need to submit their entire data set if only a portion of the data was used in the reported study.

If your submission does not contain these data, please either upload them as Supporting Information files or deposit them to a stable, public repository and provide us with the relevant URLs, DOIs, or accession numbers. For a list of recommended repositories, please see <https://journals.plos.org/plosone/s/recommended-repositories>.

If there are ethical or legal restrictions on sharing a de-identified data set, please explain them in detail (e.g., data contain potentially sensitive information, data are owned by a third-party organization, etc.) and who has imposed them (e.g., an ethics committee).

Please also provide contact information for a data access committee, ethics committee, or other institutional body to which data requests may be sent. If data are owned by a third party, please indicate how others may request data access.

**Response:** Thank you. We have included a minimal data set as part of our re-submission.

5. Your ethics statement should only appear in the Methods section of your manuscript. If your ethics statement is written in any section besides the Methods, please delete it from any other section.

**Response:** We have removed our ethics statement from the declarations section. It is now only mentioned under methods.

Please include captions for your Supporting Information files at the end of your manuscript, and update any in-text citations to match accordingly. Please see our Supporting Information guidelines for more information: <http://journals.plos.org/plosone/s/supporting-information>.

**Response:** Thank you. We have added captions/titles for our supporting information following the references.

If the reviewer comments include a recommendation to cite specific previously published works, please review and evaluate these publications to determine whether they are relevant and should be cited. There is no requirement to cite these works unless the editor has indicated otherwise.

**Response:** Thank you. We have included suggested references only where appropriate.

**REVIEWER 1**

Data Policy: Plos One's data policy does not permit author contact to be the only source of the data set in most cases. This study does not, under my reading of the policy, meet any of the exceptions. The full data set should be uploaded to an open data repository.

**Response:** Thank you for the careful review of policies. We have uploaded the de-identified raw quantitative data as part of our revisions.

Inadequate literature review. Other researchers have conducted qualitative and quantitative investigations into the impact of ethnicity and gender on respirator fit. These works are a) not mentioned in this paper and b) the originality / importance of this data are not established in relevance to prior work. While authors are correct sex has been more extensively studied than ethnicity, they are incorrect that ethnicity has not been much studied (lines 318-319). I would recommend the authors review the following literature on both gender and ethnicity:

--- Prospective observational study of gender and ethnicity biases in respiratory protective equipment for healthcare workers in the COVID-19 pandemic by C. Carvalho et al 2021
--- The influence of gender and ethnicity on facemasks and respiratory protective equipment fit: a systematic review and meta-analysis by J Chopra et al 2021

--- The effect of N95 designs on respirator fit and its associations with gender and facial dimensions by Hasni et al 2021
--- Respirator Fit and Facial Dimensions of Two Minority Groups by W Brazile et al 1998
--- The Effect of Subject Characteristics and Respirator Features on Respirator Fit by Z Zhuang et al 2005
--- The Effect of Gender and Respirator Brand on the Association of Respirator Fit with Facial Dimensions by R. Oestenstad et al 2007
--- P2/N95 filtering facepiece respirators: Results of a large-scale quantitative mask fit testing program in Australian health care workers by M Milosevic et all 2021
--- 316 Fit of N95 filtering facepiece respirators influenced by gender, design of facepiece, and activity engaged in use by Glass et all 2013

--- The PPE Pandemic: Sex-Related Discrepancies of N95 Mask Fit by L Christopher 2021
--- Evaluation of a Large-Scale Quantitative Respirator-Fit Testing Program for Healthcare Workers: Survey Results by Wilkinson et al 2010

**Response:** We thank the reviewer for this important observation and have revised the discussion to acknowledge prior work, not already cited, examining the influence of gender and ethnicity on respirator fit. Several studies report higher fit test failure rates among women and ethnic minority groups, particularly Asian healthcare workers, while others attribute fit primarily to individual facial dimensions and respirator design. These mixed findings underscore the importance of conducting population-specific studies, as respirator fit reflects both individual and group-level anthropometric variation. In light of Canada’s increasingly diverse healthcare workforce, we argue that locally derived evidence is essential to ensure respirator design and fit testing protocols adequately meet the needs of all healthcare workers.

Although this study was unable to obtain sufficient participants for statistical significance, I do believe the preliminary quantitative fit test findings are interesting and may potentially be of benefit to the scientific community. I am not sure that they warrant an original full paper - but if there is a brief report section, this would be a perfect fit. The significance of this paper, and it’s publishability, will depend partly on what it contributes in relation to existing literature (see point 2).

**Response:** We appreciate the reviewer’s comment and acknowledge the limitation of our sample size. However, we believe the preliminary findings remain valuable, as they provide early evidence on respirator fit in relation to gender and ethnicity within a Canadian healthcare workforce. As outlined in our revised discussion, this work contributes to the limited literature on population-specific respirator fit and underscores the importance of generating locally relevant data. To our knowledge, none of the existing studies on gender and ethnicity and respirator fit have been conducted in Canada, where the healthcare workforce is increasingly diverse; thus, locally derived evidence is essential to inform respirator design and fit testing protocols.

I would recommend the authors be more upfront / explicit about the inadequate sample size in the abstract and introduction. There is repeated mention of the study not meeting feasibility criteria. I would suggest changing the language to explicitly discuss the small sample size and reasons for this limitation. I believe the "feasibility" language currently used hides the utility of the study. Also, the current language is somewhat misleading - as this isn't a study to see if a method is feasible, as the method is well established in both scientific and industrial use.

**Response:** Thank you for this note. We have revised our study abstract and conclusion to explicitly state the limitations related to the sample size. Below is the revised text:

From the Abstract: “This study was limited by a small sample size, as COVID-19 pandemic restrictions prevented adequate recruitment to detect differences between groups.”

From the Conclusion: “This study was conducted during the COVID-19 pandemic, when restrictions on in-person research limited recruitment and resulted in a small sample size. While this precluded statistical comparisons between groups, the preliminary findings provide important insights into gender- and ethnicity-related variation in respirator fit among Canadian healthcare workers.”

The analysis of qualitative survey findings is lacking. If the qualitative findings are not robust enough to be useful, I would suggest leaving them out of the paper and instead making them available in the dataset for interested researchers. Otherwise, discussion and analysis are needed.

**Response:** Thank you for this note. Our intention in including the qualitative survey was to describe the experiences of healthcare workers with respirator fit, comfort, and impact on well-being. We agree that the qualitative data are descriptive and preliminary and have revised the manuscript to make this purpose explicit. We have expanded the discussion of these findings to highlight how they complement the quantitative results and underscore the need for further in-depth qualitative research. We believe retaining these data adds value by situating the fit test results within the lived experiences of HCWs, particularly as issues of comfort and psychological burden are central to understanding respirator use.

**REVIEWER 2**

I have reviewed the above manuscript, which addresses an important and timely question about the fit, comfort, and breathability of N95 respirators among healthcare workers of diverse genders and ethnicities. The work is relevant to occupational health, equity in PPE design, and pandemic preparedness. The mixed-methods approach is appropriate and offers valuable context to the quantitative fit test results. However, several areas would benefit from clarification and additional detail before the manuscript can be considered for publication.

**Response:** Thank you for taking the time to review our manuscript and provide detailed feedback. We sincerely appreciate the opportunity to revise and improve our manuscript.

Abstract (lines 23–48)
Clarify why the feasibility target was set at 100 participants, and what the reduced sample size means for interpreting both quantitative and qualitative results. Consider adding a sentence on whether this impacts the power to detect differences.

**Response:** Thank you for this comment. We have revised our abstract to explicitly state the small sample size and insufficient power to detect statistically significant differences between groups and have added the rationale for “100 participants” to Table 1.

Background (lines 62–96)
The background section is strong, but the reliance on pre-pandemic anthropometric data references (e.g., 2005) should be supplemented with more recent post-COVID PPE design literature.

**Response:** Thank you for the kind words! We have included more recent references in our introduction (lines 100-102) . We have also included a more detailed analysis of these studies in relation to our work in the discussion (lines 437-447).

If any Canadian-specific anthropometric studies exist, briefly mention them to strengthen the rationale for conducting this work in Canada.

**Response:** Based on comments from the other review, we have made modifications in our introduction (lines 100-102) and in our discussion (lines 437-447) to highlight the existing body of work on this topic, the lack of Canadian data, and how our study fills an important gap.

Methods (lines 106–198)
More detail is needed on how the “statistics-by-theme joint display” was created—did integration involve iterative coding, and how were disagreements resolved?

**Response:** We thank the reviewer for this suggestion. We have added further detail in the Methods to clarify how the “statistics-by-theme joint display” was created. Specifically, integration of the quantitative and qualitative findings was conducted through an iterative process in which authors independently reviewed the descriptive survey responses and then collaboratively organized the findings into a joint display to highlight points of convergence and divergence. Disagreements were resolved through discussion until consensus was reached. This approach ensured that both data sources were represented transparently and that the joint display accurately reflected the descriptive nature of the qualitative findings alongside the quantitative results. These changes are reflected in lines 207-212.

Justify the requirement that 50% of participants be non-White or have other specific characteristics. Was this based on effect size estimation, diversity representation targets, or prior literature?

**Response:** We appreciate the reviewer’s request for clarification. The requirement that at least 50% of participants be non-White was not based on effect size estimation but was instead established a priori to ensure adequate representation of participants from diverse ethnic groups. Prior literature has demonstrated that respirator fit may vary across ethnic groups, with Asian and other non-White healthcare workers experiencing higher fit failure rates compared to White workers. Without intentional recruitment, there is a risk that studies conducted in Canada would overrepresent White participants, limiting the ability to explore these equity-relevant differences. Our sampling requirement was therefore designed to align with diversity representation targets, to generate more inclusive and locally relevant evidence. We have clarified this in lines 177-181.

Explain why no exploratory non-parametric statistics (e.g., Mann–Whitney U tests) were used, as these can be informative even in small samples.

**Response:** We thank the reviewer for this suggestion. We considered the use of exploratory non-parametric tests such as Mann–Whitney U; however, given the small sample size even non-parametric analyses would have had extremely limited interpretability and risked overstating differences. Instead, we chose to present descriptive statistics and qualitative findings in parallel, which we believe more appropriately reflect the preliminary and exploratory nature of the study. This is described in lines 200-202.

Indicate whether fit testers were blinded to participant ethnicity and gender to reduce potential observer bias.

**Response:** We thank the reviewer for raising this important point. Fit testers were not blinded to participant gender and ethnicity; however, data on these characteristics were collected separately from the fit testing procedure and were not available to testers at the time of assessment. As fit testing is a standardized, protocol-driven process that relies on quantitative measurement rather than subjective judgment, we believe the risk of observer bias influencing outcomes was minimal.

The exclusion criteria should be reported precisely.

**Response:** We thank the reviewer for this suggestion and have clarified the exclusion criteria in the Methods section. The only exclusion criterion was being unable to safely complete a PortaCount fit test, for example, due to pre-existing respiratory conditions or other medical or safety concerns. This has now been explicitly reported to ensure clarity and transparency.

Results – Feasibility (lines 200–231)
The feasibility shortfall is described, but more interpretation is needed. Were these challenges purely pandemic-related, or do they indicate systemic issues in recruiting HCWs for PPE research?

**Response:** Thank you for this comment. The primary challenges to recruitment were pandemic-related, as research activities were restricted, HCWs were under extreme clinical demands, and site access for study personnel was limited. These extraordinary circumstances significantly constrained our ability to recruit, rather than indicating a systemic barrier to engaging HCWs in PPE research. Indeed, the high consent rate among eligible participants (90.2%) suggests strong willingness to participate when conditions allow.

Consider quantifying the recruitment rate per week before and after the study amendment to better illustrate the impact of additional sites.

**Response:** Thanks for this note. Details about the additional number of people we were able to recruit following the amendment are available in Figure 1 where we list participants by site.

Results – Quantitative Fit Test (lines 233–259)
For anthropometric results, consider providing percentage differences to make the magnitude of differences more interpretable.

**Response:** We thank the reviewer for this suggestion. While we considered presenting anthropometric results as percentage differences, we elected not to do so, as the small sample size limits the interpretability of such comparisons and may give a misleading impression of precision. Instead, we have retained absolute measurements and descriptive statistics, which more accurately reflect the preliminary and exploratory nature of this dataset. We believe this approach provides clarity without overstating the magnitude of differences.

The finding that 75% of participants fall outside the NIOSH bivariate panel range is important—state briefly in Results what this implies for respirator design testing standards.

**Response:** Thank you for this suggestion. We have revised the Results to briefly state the implications of 75% of participants falling outside the NIOSH bivariate panel range. Specifically, we note that this suggests current respirator design and testing standards, which rely heavily on the NIOSH panel, may not adequately reflect the facial anthropometry of a diverse healthcare workforce. This revision can be seen in lines 319-321. We are aware of more recent ISO standards for fit testing that involve more measurements. This is the focus of an ongoing followup study.

Results – Qualitative (lines 261–297)
Tables 4a and 4b should include scale anchors (e.g., 0 = poor, 4 = excellent) directly in the table for clarity.

**Response:** Thank you for this suggestion. We have added the scale anchors to tables 4a and 4b.

Discussion (lines 299–360)
The comparison to prior literature is solid, but the discussion should explicitly address whether the trends observed (e.g., gender/ethnic disparities in fit) are likely to persist outside pandemic surge conditions.

**Response:** We thank the reviewer for this valuable suggestion. We have revised the Discussion to explicitly consider whether the trends observed in our study are likely to persist outside of pandemic surge conditions. While recruitment challenges were clearly pandemic-related, the observed disparities in fit by gender and ethnicity are consistent with prior literature from non-pandemic contexts, suggesting that these patterns are not unique to COVID-19 surge conditions. These changes are reflected in lines 473-475.

Acknowledge the absence of participants with religious head coverings or facial hair, as this limits the generalizability to all HCW subgroups.

**Response:** Thank you. We have explicitly stated this limitation in the discussion.

Conclusion (lines 362–368)
Strengthen the policy recommendations by explicitly calling for updates to the NIOSH bivariate panels and Canadian standards to incorporate gender and ethnic diversity in respirator design, along with specific recommendations for manufacturers.

**Response:** Thank you for this suggestion. We have revised the Conclusion to strengthen the policy implications of our findings. Specifically, we now highlight the need for updates to Canadian standards to better reflect gender and ethnic diversity in respirator design and fit testing protocols. In addition, we have added explicit recommendations for manufacturers to incorporate diverse anthropometric data into respirator development, with the goal of ensuring more equitable protection for all healthcare workers. These changes were made in lines 498-501.

Figures and Tables
Include sample sizes (n) in figure legends for transparency.

**Response:** Thank you for this suggestion. We have included these details in the figure caption which will appear alongside the figure in the final publication.

In Figure 2, annotate the proportion of participants outside the panel range for emphasis.

**Response:** Thank you for this suggestion. We have included these details in the figure caption which will appear alongside the figure in the final publication. The figure caption reads as follows:

“The facial measurements of the 36 included HCWs are displayed alongside the NIOSH bivariate panel. These panels correspond to a range of face widths (120.5 – 158.5 mm) and lengths (98.5 – 138.5mm) and recommend number of participants to include from each panel when testing new respirators. Twenty-seven of the thirty-six (75%) included HCWs in this study were out of range, demonstrating differences in this sample and the testing standards. “
